# A Preliminary Assessment of the Potential Health and Genetic Impacts of Releasing Confiscated Passerines Into the Wild: A Reduced-Risk Approach

**DOI:** 10.3389/fvets.2021.679049

**Published:** 2021-10-11

**Authors:** Cláudio E. F. Cruz, Gustavo R. Funkler, André L. S. Zani, Paulo G. C. Wagner, Inês Andretta, Luciano N. Segura, Nelson J. R. Fagundes

**Affiliations:** ^1^Centro de Estudos em Manejo de Aves Silvestres, Faculdade de Veterinária, Universidade Federal do Rio Grande do Sul, Porto Alegre, Brazil; ^2^Programa de Pós-Graduação em Ciências Veterinárias, Faculdade de Veterinária, Universidade Federal do Rio Grande do Sul, Porto Alegre, Brazil; ^3^Laboratório Porto Belo, Porto Alegre, Brazil; ^4^Programa de Pós-Graduação em Genética e Biologia Molecular, Instituto de Biociências, Universidade Federal do Rio Grande do Sul, Porto Alegre, Brazil; ^5^Centro de Triagem de Animais Silvestres, Instituto Brasileiro do Meio Ambiente e dos Recursos Naturais Renováveis, Porto Alegre, Brazil; ^6^Laboratório de Ensino Zootécnico, Faculdade de Agronomia, Universidade Federal do Rio Grande do Sul, Porto Alegre, Brazil; ^7^Museo de La Plata, Sección Ornitología, Universidad Nacional de La Plata, La Plata, Argentina; ^8^Programa de Pós-Graduação em Biologia Animal, Instituto de Biociências, Universidade Federal do Rio Grande do Sul, Porto Alegre, Brazil

**Keywords:** seized songbirds, rehabilitation and release, mycoplasma, outbreeding depression, wild bird management, wildlife policy, animal welfare

## Abstract

The illegal capture and trade of wild birds have long been threats to biodiversity. The rehabilitation and release of confiscated animals may be a useful conservation tool in species management. However, differences between populations regarding health (e.g., different pathogens) and adaptation (e.g., local adaptation) must be taken into account, since both can negatively impact the recipient population. In this pilot study, we used two of the most illegally trafficked Brazilian wild passerine species, namely the red-crested cardinal (*Paroaria coronata*) and green-winged saltator (*Saltator similis*) as case studies and assessed some of the health threats that the release of confiscated passerines may pose to free-living birds. We also investigated the level of difference in mitochondrial genetic structure among populations living in different ecoregions. Blood, feces, and oropharyngeal swabs from confiscated (*n* = 115) and free-living (*n* = 120) passerines from the release sites were tested for the Newcastle disease virus, *Salmonella* spp., and *Mycoplasma gallisepticum*. These are considered major avian diseases by the Brazilian National Avian Health Program. We analyzed mtDNA to study the difference in genetic structure between populations using samples from 127 free-living passerines. We found no evidence of the Newcastle disease virus or *Salmonella* spp. in confiscated or free-living passerines from either species. However, the levels of infection with *M*. *galissepticum* detected in our study for red-crested cardinals and green-winged saltators calls for a high degree of caution in captive release programs. The difference in genetic structure between populations occurring in different regions was low, and was not significant between those from the Pampa/Subtropical Grasslands region. These results suggest that it may be possible to establish a cost-effective and sensitive protocol for releasing confiscated songbirds, provided that further genome-wide studies indicate that the functional genetic diversity among (at least some of the) populations is also low.

## Introduction

Illegal trade, poaching, habitat loss, and pollution are the main causes of the decline in wild bird populations in Brazil, and other developing countries with vast species diversity (1–4). The illegal wildlife trade has increased dramatically over the past decade, along with enforcement efforts aimed at mitigating this threat (5, 6). Current guidelines for the management of confiscated wild birds include the option of humanely killing animals from species with low conservation value (7). This policy has been justified on the grounds that confiscated birds a) may harbor pathogens that will affect the wild population (8–10) and b) usually come from an unknown parental population, so their release into another population may lead to outbreeding depression (11, 12). In both cases, there may be a negative impact on the wild population. The decision to release confiscated wild birds should be taken on a case-by-case basis and be performed according to conservation guidelines. These, in turn, should be based on genetic and health data as well as other studies (6, 7, 9). However, this information is scarce and there is limited conservation evidence on the subject.

The Brazilian poultry industry has considerable socioeconomic importance and the National Avian Health Program (*Plano Nacional de Sanidade Av*í*cola*—PNSA) establishes official measures for the prevention, control, and surveillance of diseases mainly associated with poultry, i.e., salmonellosis, mycoplasmosis, and Newcastle disease (13). Among the numerous conditions that can affect wild birds (14), PNSA-recognized pathogens can impact avian health in both free-living and commercial flocks (9, 10, 14–16). Whenever the decision is made to return confiscated individuals to the wild, it is crucial to avoid endangering the health, behavioral repertoire, and genetic and conservation status of wild populations of the species, as well as ensure the welfare of the released animals (6–10). In this study, we explored these issues by generating preliminary genetic and health-related data from wild red-crested cardinals (*Paroaria coronata*) and green-winged saltators (*Saltator similis*), two of the most illegally trafficked wild bird species in Brazil (17, 18). More specifically, we aimed at addressing to two fundamental questions: are there significant differences in the prevalence of PNSA-recognized pathogens between confiscated and free-living passerines from the release sites, and is the difference in mitochondrial genetic structure among free-living populations of these species significant? Finally, we discuss the potential impacts of a release program for confiscated conspecific passerines of both species.

## Materials and Methods

### Sampling Areas

**Figure 1:**
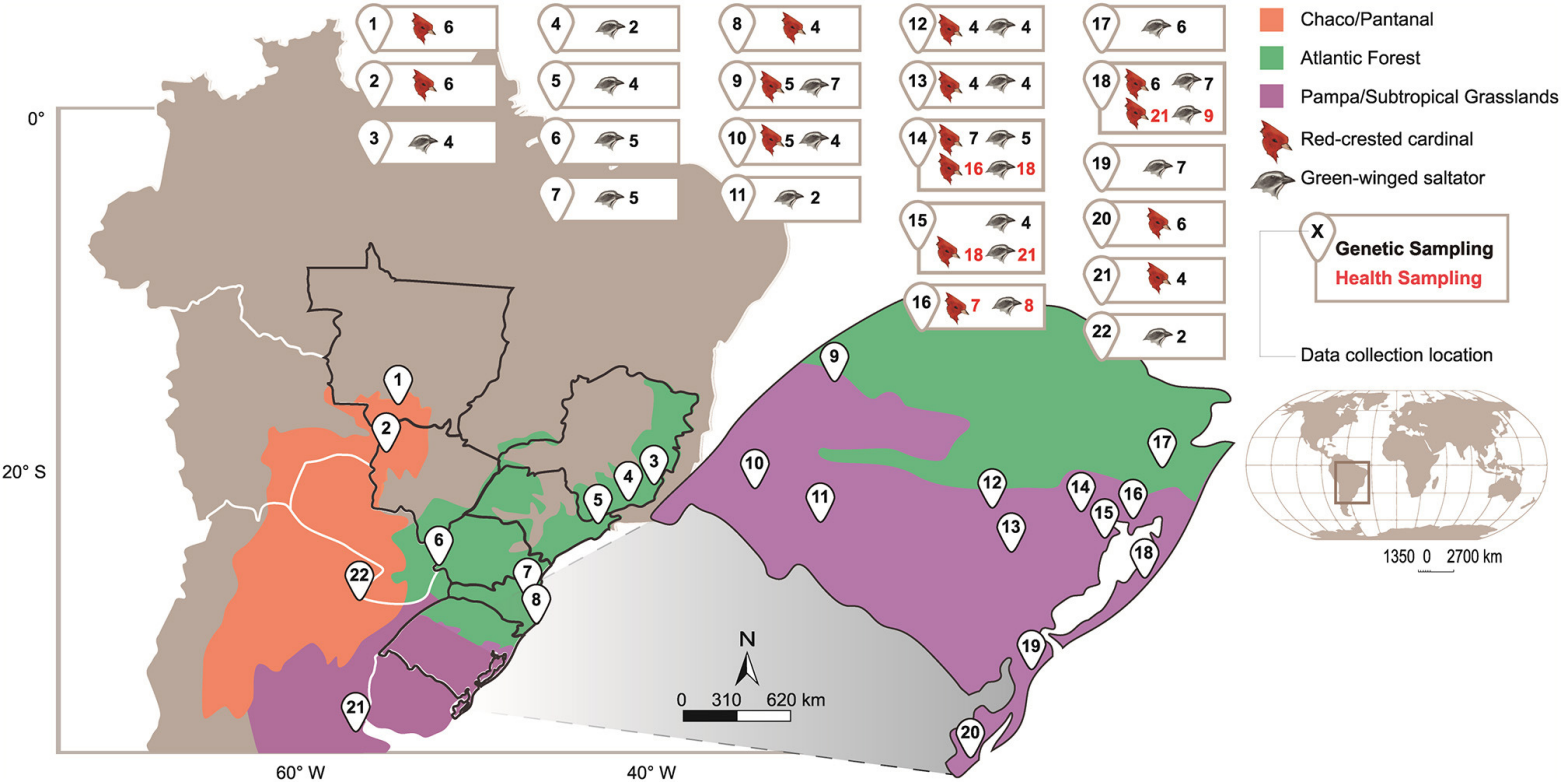
Geographical representation of sampled areas with the respective number of samples.

Sampling areas were selected to include ecoregion domains representative of the species' typical distribution, mostly in the state of Rio Grande do Sul (RS), as well as strategic areas across the species distribution (Figure 1). Green-winged saltators were sampled in the forest fragments of two different regions: Pampa/Subtropical Grasslands (PSGrasslands) and Atlantic Forest (AForest). Red-crested cardinals were sampled in semiopen areas in three different regions: PSGrasslands, Chaco/Pantanal (CPantanal), and AForest (anthropic deforested areas). These ecoregions were used as proxies for the ecological populations from which the genetic structure was tested. PSGrasslands include the Uruguayan Savanna, the Humid Pampa, Espinal, and the Southern Cone Mesopotamian Savanna formations. The CPantanal area includes the Humid Chaco, the Dry Chaco, and Pantanal formations, while the AForest includes Alto Paraná Atlantic Forests, Araucaria Moist Forests, Serra do Mar Coastal Forests, and Bahia Coastal Forests (19). To distribute financial resources across multiple collection sites, fieldwork efforts were based on an average capture rate. Based on similar studies (20–23), we aimed to achieve a sample size of about 5–10 individuals. However, in sites where the species abundance was low, the sample size was lower. For genetic analyses, we included sequences from GenBank (Bolivia *n* = 1, Boracéia *n* = 1, Corrientes *n* = 2, Mato Grosso *n* = 1) and five additional red-crested cardinal samples from Buenos Aires, Argentina (the southernmost species distribution limit).

### Health-Related Tests

We tested for selected pathogens in groups of free-living and confiscated conspecific passerines from both species. Samples from free-living passerines were obtained from the recipient populations in the release areas located no further than 200 km away from the laboratory facilities, to ensure they could be delivered to the laboratory on the same day of collection (Supplementary Table 1). Following the standard guideline (5, 9) in rehabilitation and release, the disease screening included both confiscated and free-living conspecific wildlife from the reception area. These areas were also selected to accommodate long-term post-release monitoring. Due to budgetary constraints, we decided to test a 10%-sample out of the total confiscated flock that was quarantined and rehabilitated in 1 year, and a similar number of free-living birds. The samples were kept refrigerated during transport to the laboratory. Samples from confiscated passerines were obtained from birds kept in the triage centers (*Centro de Triagem de Animais Silvestres*—CETAS, IBAMA and Fundação Zoobotânica—FZB/RS). These passerines were of unknown origin since they could have been illegally captured anywhere across their distribution ranges. Usually, in these centers, large numbers of confiscated passerines of the same or different species are kept in dozens of contiguous cages. Precise information regarding the duration of captivity before apprehension is not available. We estimated that most of the cage-conditioned confiscated passerines had been kept several months in captivity, although most freshly caught (based on their wild behavior) confiscated passerines had been captive for up to 7 days. After sampling and undergoing clinical and behavioral history assessments, the passerines were placed with IBAMA rehabilitation/release projects or maintained in captivity.

Microbiological, serological, and molecular tests were performed at the Porto Belo Laboratory, accredited by the Brazilian Ministry of Agriculture, Livestock Farming and Food Supply (13) to perform official diagnostic tests for the PNSA. The hemagglutination-inhibition (HI) test for anti-NDV antibodies was performed in 96-well U-bottom microtitration plates using fresh red cells from SPF birds collected in Alsever's solution and washed in PBS. Four hemagglutination units (UHA) of NDV LaSota viral suspension were prepared immediately before running the test. The tested sera were previously diluted in PBS and added to the plates (50 μL/well) in 1:2 to 1:1024 dilutions and tested in duplicate. Afterward, 50 μL of four UHA viral suspensions were added to each dilution of the serum and incubated for 30–45 min at RT. Next, 50 μL of the 1% red blood cell suspension was added and the plate was incubated for another 30–45 min at RT. Each test included positive and negative controls, apart from the control titration of four UHA and the control of red blood cells. The titer level was expressed as the reciprocal of the highest dilution that completely inhibited hemagglutination (24). The methods described in NI 126 (25) were used to test for *Salmonella* spp., after replacing the BHI broth with 1% buffered peptone water for non-selective enrichment (a sample-broth proportion of 1:9) and incubation at 36 ± 1°C for 18–24 h. The enriched broth (1 and 0.1 mL, respectively) was then inoculated in Tetrathionate Broth (supplemented with 0.1% brilliant green and iodine or iodine solution) and Rappaport–Vassiliadis broth, and incubated at 42.5 ± 0.5°C for 18–24 h. It was then streaked on Hektoen Enteric and MacConkey Agar and incubated at 36 ± 1°C by 18–24 h. At the Oswaldo Cruz Foundation, World Health Organization protocols (26) were used to confirm and characterize suspected colonies with specific antisera. Serum samples were analyzed for anti-MG antibodies using rapid plate agglutination (RPA). The test was carried out by mixing 25 μL commercial MG-antigens (INATA Biologic Products, Uberlândia, MG, Brazil) with 25 μL serum samples for 2 min at RT (27). Real-time polymerase chain reactions were performed, using a Taqman-labeled probe to detect *M*. *gallisepticum* (commercial kit MG—NewGene®, Simbios Biotecnologia, Cachoeirinha, RS, Brazil) DNA (28). As described previously (28, 29), CTA GAG GGT TGG ACA GTT ATG−3′, GCT GCA CTA AAT GAT ACG TCA AA−3, and CAG TCA TTA ACA ACT TAC CAC CAG AAT CTG–(MGB)−3′ primers and probes were used to target the MG lipoprotein gene. Positive results were sent to Simbios Biotechnology laboratory for confirmation via real-time and conventional PCR. Serum samples from six captive conspecific passerines, vaccinated against MG and NDV, served as controls for MG-qPCR and NDV-HI validation. The samples were tested before and after vaccination as negative and positive controls, respectively. Live MG (MYCOVAX®TS-11, MERIAL, strain TS-11) and NDV vaccines (MERIAL, strain La Sota 004/16, ND1873) (Boehringer Ingelheim Saúde Animal, Paulínia, SP, Brazil) were administered via eye drop, at one drop per bird, 21 days apart. Boosters consisted of one eye drop per bird, 28 days apart for both live MG (BIOCAMP, Camp VacMG-F) and NDV vaccines (BIOVET, New-Vacin, La Sota). An additional oil inactivated NDV vaccine (BIOVET, New-BRONK-VET, virus B1 La Sota, minimum title before inactivation 10^5,3^ DIOE_50_) (BIOVET, Vargem Grande Paulista, SP, Brazil) was administered intramuscularly 53 days after the last NDV eye-drop vaccine. This protocol was based on a previous study of NDV vaccination in wild birds (30). Details of the disease screening protocol are provided elsewhere (31).

### Genetic Analysis

To understand the genetic structure and diversity of the two species, we compared passerines from the PSGrasslands region, where both species are relatively abundant, with red-crested cardinals from CPantanal and green-winged saltators from the AForest area. There were few samples of red-crested cardinal and green-winged saltators from the AForest and CPantanal regions, respectively, because these species do not commonly occur in these areas. This strategy also allowed us to compare two regions with similar vegetation patterns (PSGrasslands vs. CPantanal, in the case of red-crested cardinals) to two areas with different patterns (PSGrasslands vs. AForest, for green-winged saltators). DNA was extracted from blood on FTA cards using a PureLink Genomic DNA Mini Kit (Invitrogen, Carlsbad, California, USA), and a fragment of subunit 2 of mitochondrial NADH dehydrogenase (*ND2*) was amplified using the same PCR protocol for both species. The reaction was performed with 20 ng/μl of DNA, 1x PCR Buffer (Invitrogen, Carlsbad, California, USA), 3.5 mM MgCl2, 0.2 mM dNTPs, 0.2 pmol/μl of each primer, and 0.04 U/μl of Taq Platinum DNA Polymerase (Invitrogen, Carlsbad, California, USA). The primers used were MetL, as described previously (32), sequence 5′-AAGCTATCGGGCCCATACCCG-3′) and RND2A (this study, sequence 5′-CCTGAGTTGCATTYAGGGG-3′). The PCR protocol was as follows: 94°C for 2 min, 35 cycles of 94°C for 30 s, 59°C for 30 s, 72°C for 60 s, and a final extension of 72°C for 8 min. Amplification was confirmed using electrophoresis in a 1% agarose gel. The amplified products were enzymatically purified with exonuclease I (GE Healthcare, Chicago, Illinois, USA) and Sanger sequenced by ACTGene Inc. (Alvorada, RS, Brazil).

### Data Analysis

Pearson's chi-square test was used to assess the association between categorical variables. The 95% Confidence intervals (CI) were obtained using a one-sample proportion test. The analyses were conducted with Minitab v. 18 software (State College, Pennsylvania, USA. http://www.minitab.com), at a 0.05 significance level. Responses (health-related) were also expressed using descriptive statistics. Regarding molecular data, DNA sequences were assembled and aligned using the default settings in Geneious v.10.2.3 (https://www.geneious.com) and visually checked in MEGA X v.10.0.0 (33). The DnaSP v.6.12.03 program (34) was used to define the different haplotypes, whose evolutionary relationships were represented in a median-joining network (35), and estimated with PopART software (http://www.popart.otago.ac.nz). Standard genetic diversity indices, including Tajima's D (36) and Fu's FS (37) neutrality tests were estimated using Arlequin v.3.5.2.2 software (38). This software was also used to quantify the level of genetic structure via hierarchical and non-hierarchical analyses of molecular variance (AMOVA) (39). Hierarchical AMOVA was performed for each species and all three collection sites (PSGrasslands, AForest, and CPantanal). Non-hierarchical AMOVA was only conducted for PSGrasslands, to estimate the level of structure within one region, using a comparable sample strategy for both species. Finally, to understand the past demography of both species, we used BEAST v.2.6.1 software to generate Bayesian skyline plots (BSP) (40) for the total population and the main regions of occurrence of each species (41). A total of 10,000,000 MCMC steps were used, with sampling every 1,000 steps. The initial 10% of the run was discarded as burn-in. A partition scheme was applied, allowing each codon position to have a different substitution model, which was estimated using MEGA X v.10 (33). The Tracer v.1.7.1 program was used to check sampling sufficiency to construct the BSP (42). The molecular substitution rate for the ND2 gene was calculated as previously described (43), assuming “calibration set 2” for a 45 g bird (44, 45).

## Results

### Health-Related Tests

The serological study revealed no antibodies against Newcastle disease, irrespective of the origin of the bird. *Salmonella* spp. isolation resulted in only one positive sample: *S*. *enterica* serovar Cerro from a free-living red-crested cardinal. Two tests were used to detect *M. gallisepticum* (MG). The RPA test indicated positive MG results for 22% of samples from free-living passerines (15–30% CI), although none were confirmed by PCR. In the confiscated bird sample, the seroprevalence (51%, 42–61% CI) was higher than the molecular prevalence (14%, 8–22% CI) (data on the PNSA-recognized tests and samples from free-living and confiscated passerines of both species, as well as anti-NDV antibody titers in control birds and confidence intervals, are presented in Supplementary Table 2).

### Genetic Structure and Diversity

**Figure 2:**
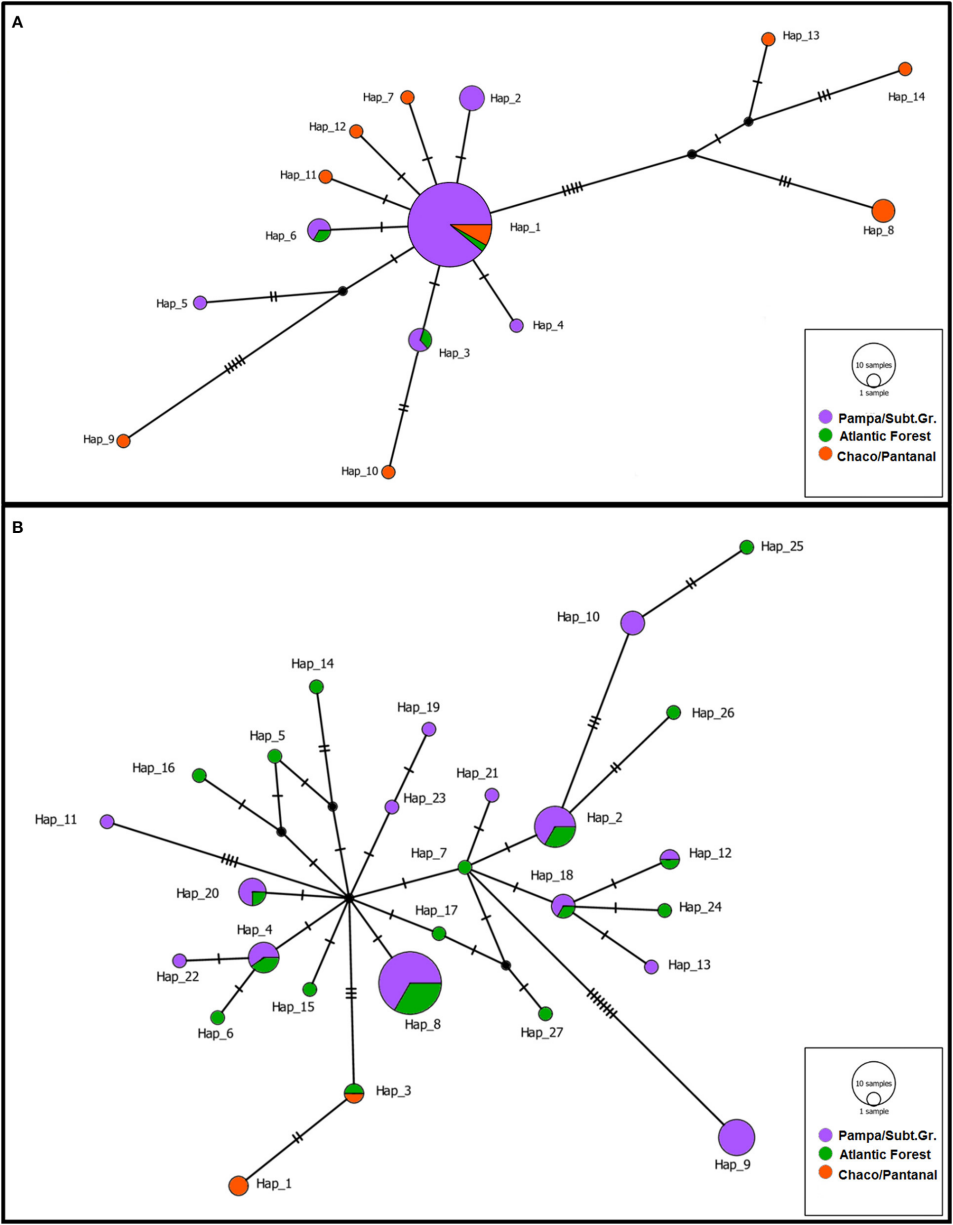
Median-joining network for the haplotypes of red-crested cardinals **(A)** and green-winged saltators **(B)**. Circle size is proportional to the number of birds in the sample with that haplotype. Transversal lines represent mutational steps.

DNA sequence analysis resulted in the alignment of 977 bp and 810 bp for red-crested cardinals and green-winged saltators, respectively. All new sequences were deposited in the GenBank (Supplementary Table 3). Genetic diversity indices for both species in all the sampled regions are shown in Supplementary Table 4. Only free-living passerines were included in the genetic analysis. Overall, green-winged saltators exhibited greater genetic diversity than red-crested cardinals for both the whole sample and the PSGrasslands region. However, there were contrasting patterns between species when the two major regions of occurrence were compared. While green-winged saltators showed comparable levels of diversity in the PSGrasslands and AForest, the diversity for red-crested cardinals in the former area was only a fraction of that observed in the latter. This was also evident in the haplotype network (Figure 2). For both species, the sample size in regions of minor occurrence (the AForest for red-crested cardinals and the CPantanal for green-winged saltators) was too small for thorough genetic diversity characterization.

The haplotype networks for the two species (Figure 2) also suggest a relatively low degree of genetic structure between regions for both species. Indeed, when all regions are considered, 26.18% of the total genetic variance (Φ_*CT*_ = 0.2618; *P* = 0.040) for red-crested cardinals occurred among regions, whereas the respective value for green-winged saltators was only 12.94% (Φ_*CT*_ = 0.1294; *P* < 0.001). On the other hand, most genetic variation occurred within populations, reaching 65.17% (Φ_*ST*_ = 0.3483; *P* < 0.001) and 82.72% (Φ_*ST*_ = 0.1728; *P* = 0.011) for red-crested cardinals and green-winged saltators, respectively. The genetic structure among populations within regions accounts for the remaining portion of genetic variance, but it was not significant for either species (Φ_*SC*_ = 0.1171; *P* = 0.069 and Φ_*SC*_ = 0.0499; *P* = 0.090 for red-crested cardinals and green-winged saltators, respectively). Similarly, when only the PSGrasslands region was considered, the genetic structure was not significant for either species (Φ_*ST*_ = −0.0510; *P* = 0.923 and Φ_*ST*_ = 0.0711; *P* = 0.107 for red-crested cardinals and green-winged saltators, respectively). Tajima's D and Fu's FS test statistics showed evidence of population growth in both species, though in different environments (Supplementary Table 4). The Bayesian skyline analyses corroborated these signatures, with a more recent expansion (~50,000 years ago) for red-crested cardinals in PSGrasslands, and an older expansion (~350,000 years ago) of green-winged saltators exclusive to AForest (Supplementary Figure 1).

## Discussion

Our planet is facing several challenges for biodiversity conservation, all associated with the growing human population (3, 46). Given the limited financial resources for conservation, it is understandable that non-threatened species are not considered a high priority (6, 7). However, the economic relevance of birds is not sufficiently appreciated and the economic pertinence of their ecological roles in human society is even less understood (47). The IUCN recognizes that returning confiscated animals of low conservation value to the wild may be a legitimate measure, provided there are available resources and that the process is performed per standard conservation guidelines (6, 7, 9). Alternatives to the annual, humane killing of large numbers of confiscated wild passerines (17, 18) can and should be investigated, especially considering long-term biodiversity conservation in a changing world (3, 48). Indeed, there is evidence that common species may also be susceptible to population decline (49, 50). The species studied here are among the most common in apprehensions in Brazil (17, 18), and this poses a legal and conservation challenge despite their “least concern” rating on the IUCN Red List (51). Limited genetic (52) and health data (20, 53) are available about these and other species, which are not global conservation flagships. Besides disturbing and harming the resident populations, poorly conceived projects can lead the released animals to death from starvation, undoubtedly an even worse outcome than humane killing (6, 7). Therefore, in addition to the conservation issue, questions raised here may also be relevant for animal welfare.

### Health and Disease

Although there is a long list of diseases that affect wild birds (14), we opted for investigate those addressed by the PNSA (i.e., salmonellosis, mycoplasmosis, and Newcastle disease). This choice was based on legal veterinary restrictions from specific National Regulations (9, 13), the associated economical relevance, and the scarce research on PNSA-recognized pathogens in wild passerines. Although certainly desirable, individual testing may be an unattainable goal if the numbers of confiscated birds are considered alongside the testing costs. The project's financial resources were roughly distributed in an equal manner to perform testing (genetic and pathogens), aviary construction, and bird management.

All samples tested for the presence of anti-NDV antibodies were negative in this study. Comparable results have been reported in NDV serological surveys in captive (54) and free-living wild birds (55). As noted in a previous study (30), the anti-NDV antibody titers observed in vaccinated control wild birds may have been underestimated, probably due to the species-specific method originally developed for chickens. Small volumes of blood samples obtained from passerines may limit both the production of a specific red blood cell solution and the use of duplicates in the assay. These are some of the challenges for future research. The prevalence of *Salmonella* spp. in wild birds has been attributed to exposure to environments inhabited by people and domestic animals (56). Previous studies have estimated the prevalence of *Salmonella* spp. in samples of wild birds—including passerines confiscated from illegal traffickers—at 1–7% (14, 57). Different from our expectations, only one fecal sample (0.4%) tested positive for *Salmonella* spp. culture and isolation. The positive sample contained *Salmonella enterica* serovar Cerro (Group K), a highly prevalent serovar in herds of cattle (58). Although this was also observed in our study, how it impacts the health of the free-living red-crested cardinal population is yet to be determined.

As expected, the prevalence of *M. gallisepticum* was greater after the RPA test when compared to the qPCR assay, for both free-living and confiscated birds (29, 59). Even though the seroprevalence was higher than the molecular prevalence, RPA results were highly correlated (*P* < 0.001) with those of the PCR via Pearson's chi-squared test. The qPCR-based estimate of a 14% MG prevalence in the confiscated group is similar to findings reported for other avian hosts (16, 60). The mean cycle threshold (Ct) values (Supplementary Table 2) observed in positive samples are consistent with those expected in subclinical infections (61). The high MG prevalence in confiscated birds probably reflects the poor hygiene and stressful conditions these birds are exposed to (14). Transmission of MG is largely dependent on contact between infected and susceptible hosts and therefore is facilitated under the usual overcrowding conditions that characterize the illegal wild bird trade. Although none of the positive birds in our sample showed any clinical evidence of infection, differential MG-susceptibility across bird species has been associated with both clinical and subclinical signs (14), including in wild birds (16, 62). It is well-known that most wild birds will do their best to mask signs of disease as a basic survival behavior. Furthermore, the presence of pathogens may be influenced by several factors, such as diet, environment, general health, and co-infection among others (63). These factors, alone or together, and to greater or lesser extents, all probably affect confiscated wild birds. While all avian diseases analyzed in this study have been demonstrated in the wild (14), there has been an increasing threat to the maintenance of ecosystem services and ensured environmental health (46, 48). The information included here may help counteract some epidemiological uncertainties related to the interacting effects of disease transmission, the illegal wild bird trade, the increase in wild-domestic bird interfacing, and the associated exchange between reservoirs.

### Genetic Diversity, Structure, and Implications for Outbreeding Depression

Both species in our study showed different levels of genetic diversity and different evolutionary demographic histories. Green-winged saltators exhibited greater genetic diversity overall, with older population expansion restricted to AForest populations (while the PSGrasslands population remained constant). There were similar levels of diversity between the PSGrasslands and AForest, the main regions of occurrence. On the other hand, red-crested cardinals displayed lower diversity and weaker and more recent population expansion restricted to the PSGrasslands (the CPantanal population remained constant). There was lower diversity in the PSGrasslands area when compared to the CPantanal region (Figure 2; Supplementary Figure 1; Supplementary Table 4). Among other passerines, particularly those from open areas and/or dry forests in southern South America, red-crested cardinals from the CPantanal showed high diversity at both the haplotype and nucleotide level (21, 64). Although some populations of yellow cardinal (*Gubernatrix cristata*), a highly endangered Thraupidae, exhibited high nucleotide diversity values (21), the authors used the mtDNA control region (mtDNA-CR) as a genetic marker, which evolves more rapidly than ND2 (65).

On the contrary, red-crested cardinals from the PSGrasslands area displayed low diversity for both indices. This may indicate that the population inhabiting the PSGrasslands is a recent offshoot from the CPantanal area, which may have acted as a large source population for this species. This is consistent with the exclusive signature of population expansion in the PSGrasslands region around 50,000 years ago (Supplementary Figure 1) since population growth is expected in refuge areas (66). Concerning species occurrence in the Brazilian Atlantic Forest, and other associated humid forests, green-winged saltators showed high diversity in both the PSGrasslands and AForest. Based on mtDNA coding genes, green-winged saltators from the AForest displayed the highest nucleotide diversity among forest-associated species and the fifth-highest in the PSGrasslands population. Both populations also exhibited high haplotype diversity (67–71). The exclusive signature of an ancient population expansion for green-winged saltators in the AForest region, but not in the PSGrasslands area, is curious. Most studies that detected expansion in specific populations or phylogroups suggest much more recent time scales (similar to the red-crested cardinals) (67, 68, 70, 71). Two exceptions are the greenish Schiffornis (*Schiffornis virescens*) (69) and the white-rimmed warbler (*Basileuterus leucoblepharus*) (68), which demonstrated population expansions around 150,000–300,000 years ago. The high genetic diversity and lack of a population expansion signature in the PSGrasslands region are intriguing. It is unlikely that this region served as a refuge area for green-winged saltators, which are more strongly associated with humid forests to the north, where the species shows greater genetic diversity. The lack of strong difference in genetic structure between these populations may indicate that the PSGrasslands were occupied following population expansion in the AForest area, but subsequently lost genetic diversity, which eroded the signature of an ancient expansion.

Genetic structure was weak, but significant, for both species (Figure 2). In the PSGrasslands region and associated dry forests, both the yellow cardinal (21) and the white-tipped plantcutter (*Phytotoma rutila*) (64) showed stronger structure between habitats (Φ_*ST*_~0.45). For forest species, most studies reported the presence of strongly differentiated phylogroups, either between different habitats or across the AForest (67, 70, 71). Once again, the greenish Schiffornis (69) and the white-rimmed warbler were exceptions (70), with no phylogeographic breaks across their distribution in the AForest. The lack of genetic structure in green-winged saltators is corroborated by a study that used microsatellite (SSR) loci to demonstrate that only 0.1% of total genetic variation occurred between populations distributed in different Brazilian biomes (AForest, Cerrado, Caatinga, and ecotones between them) (52). Similarly, only 3% of total genetic variation occurred among red-cowled cardinal (*Paroaria dominicana*) populations, a species closely related to the red-crested cardinal found in the Brazilian Northeast (52), and that may share a similar pattern of gene-flow. While these studies seem to point to the same direction than ours, it is important to highlight that our results must be seen as a preliminary assessment of the difference in genetic structure among populations of these species in these ecoregions. Further studies based on genome-wide (SSR or ddRADseq) markers are needed to reveal the genetic structure of these species along the whole genome and at a finer geographic scale, which could identify preferential routes for gene flow or if there are loci subjected to local adaptation, for example. Thus, in spite of the relative shallow genetic structure found in this study, especially for poulations coming from the same ecoregion, it is difficult to predict the risk of outbreeding depression by crossing individuals from populations with different genetic structure. Although genetic distance is not a good predictor of outbreeding depression (72), the latter requires different genetic adaptations to local environments that will be eroded by crossing animals from exogenous, ecologically divergent, populations (11, 12, 73). Evaluating the feasibility of releasing confiscated birds in the wild awaits for a better understanding of population connectivity among and within ecoregions.

### Concluding Remarks

Managing birds confiscated from the illegal trade is a complex and difficult issue. Although the recommendation to humanely kill wild birds belonging to species with low conservation value is justifiable (7), it is important to consider whether new information for local species could reduce the perceived risk of translocating these and other passerine species. The present study demonstrated that it may be feasible to screen confiscated passerines for several pathogens and that, although only one out of three pathogens was detected, the risk of disease transmission during handling or after release into natural environments implies a need for a systemic disease screening program. For our cases, we followed specific local veterinary regulations (8–10, 13). Confiscated passerines may be potential sources of *Mycoplasma gallisepticum* transmission to wild bird communities and commercial flocks. The MG strain variability and pathogenicity, and the difficulty in verifying that treated birds are no longer carrying MG, indicate that efforts to segregate infected birds from the uninfected ones should be made before moving birds to the site of rehabilitation (14). Besides this, systematic pathological analyses of confiscated birds that die during quarantine/rehabilitation may help identify other important conditions to be included in future testing protocols to effectively mitigate the health risk for free-living populations. Up to the present time, there have been findings linking MG to the most prevalent conditions identified in these confiscated passerines. These results were obtained through serological and molecular testing, as well as clinical and pathological (data not shown) procedures. Our study reveals an urgent need for strategic serial testing in association with a rigorous quarantine period to safeguard wild populations. Although there are clear limitations especially associated with the sample size, these preliminary data may provide the basis for future considerations and the development of a more comprehensive management approach for returning confiscated individuals of the investigated species to the wild.

The lack of strong genetic structure among ecoregions at the mtDNA level must be seen as a first setp in deciphering the evolutionary connectivity in both species, but more research will be clearly needed to allow a thorough estimation of the potential risks and benefits of releasing confiscated birds. Further research using genome-wide markers, such as SSR or ddRADseq, will be required to access genetic structure in a finer geographic scale. MtDNA is a useful genetic marker for species identification or, when difference in genetic structure among populations is strong, as a proxy for population assignment. It is cheap and convenient to type even in a few individuals. On the other hand, typing genome-wide markers is more laborious and expensive. SSR markers require that informative loci in the focal species have been identified previously, even though once such loci are characterized, typing individuals specimens can be performed relatively easily. On the other hand, ddRADseq do not require any a priori knowledge about informative loci, but is impratical to be performed in only a few specimens at a time (74, 75). In addition, other criteria could be applied to select an area for releases, such as using different regional vocal “dialects” as a proxy for the original parental population (76) of a songbird, even though the vocal plasticity exhibited by several species may be challenging (76).

The assessment of the potential impacts presented here may be a promising starting point for the reintegration of these commonly confiscated passerine species. However, these are just some of the many aspects to consider in the complex process of reintegrating confiscated wild birds into natural environments. In this sense, evaluating the carrying capacity of the release areas is an additional pre-release concern. Additionally, long-term post-release monitoring is essential for thorough characterization of the interplay between release areas, social disturbances to local wild populations, and the behavioral ecology of the released birds. Establishing a comprehensive and definitive disease screening protocol for application in these circumstances is far beyond the scope of this paper. While the results presented in this pilot study may point to future tendencies and methods, this is perhaps lifelong research. Conservation alternatives for recycling the potential ecosystem services from thousands of confiscated wild passerines may have a strategic impact on the current global scenario of changing environment and wildlife crime.

## Data Availability Statement

The raw data supporting the conclusions of this article are available as supporting material.

## Ethics Statement

The animal study was reviewed and approved by Comissão de Ética no Uso de Animais–CEUA/UFRGS.

## Author Contributions

CC and NF: conceptualization and writing (original draft). GF, AZ, and LS: data curation. AZ, IA, and NF: data analysis. CC and PW: funding acquisition and supervision. CC, GF, AZ, PW, IA, and NF: methodology. CC: project administration. CC, PW, and LS: resources. CC, GF, AZ, PW, IA, LS, and NF: visualization. All authors contributed to the article and approved the submitted version.

## Funding

This research has been funded by the CMPC Celulose Riograndense Ltda. (Project FAURGS n° 4331-1). The funders had no role in the study design, data collection, and analysis, decision to publish, or preparation of the manuscript. All the procedures described here comply with the current Brazilian laws.

## Conflict of Interest

The authors declare that the research was conducted in the absence of any commercial or financial relationships that could be construed as a potential conflict of interest.

## Publisher's Note

All claims expressed in this article are solely those of the authors and do not necessarily represent those of their affiliated organizations, or those of the publisher, the editors and the reviewers. Any product that may be evaluated in this article, or claim that may be made by its manufacturer, is not guaranteed or endorsed by the publisher.

## Supplementary Material

Supplementary Figure 1 Bayesian skyline plot for both species.

Supplementary Table 1 Dataset free-living passerines.

Supplementary Table 2 Health-related test results for free-living and confiscated passerines.

Supplementary Table 3 Dataset genetic samples.

Supplementary Table 4 Genetic diversity indices.
