# Supplementary figures and images for: A Preliminary Assessment of the Potential Health and Genetic Impacts of Releasing Confiscated Passerines Into the Wild: A Reduced-Risk Approach
Source: Front Vet Sci. 2021 Oct 11;8:679049. doi: 10.3389/fvets.2021.679049 (PMC8542797; doi:10.3389/fvets.2021.679049)

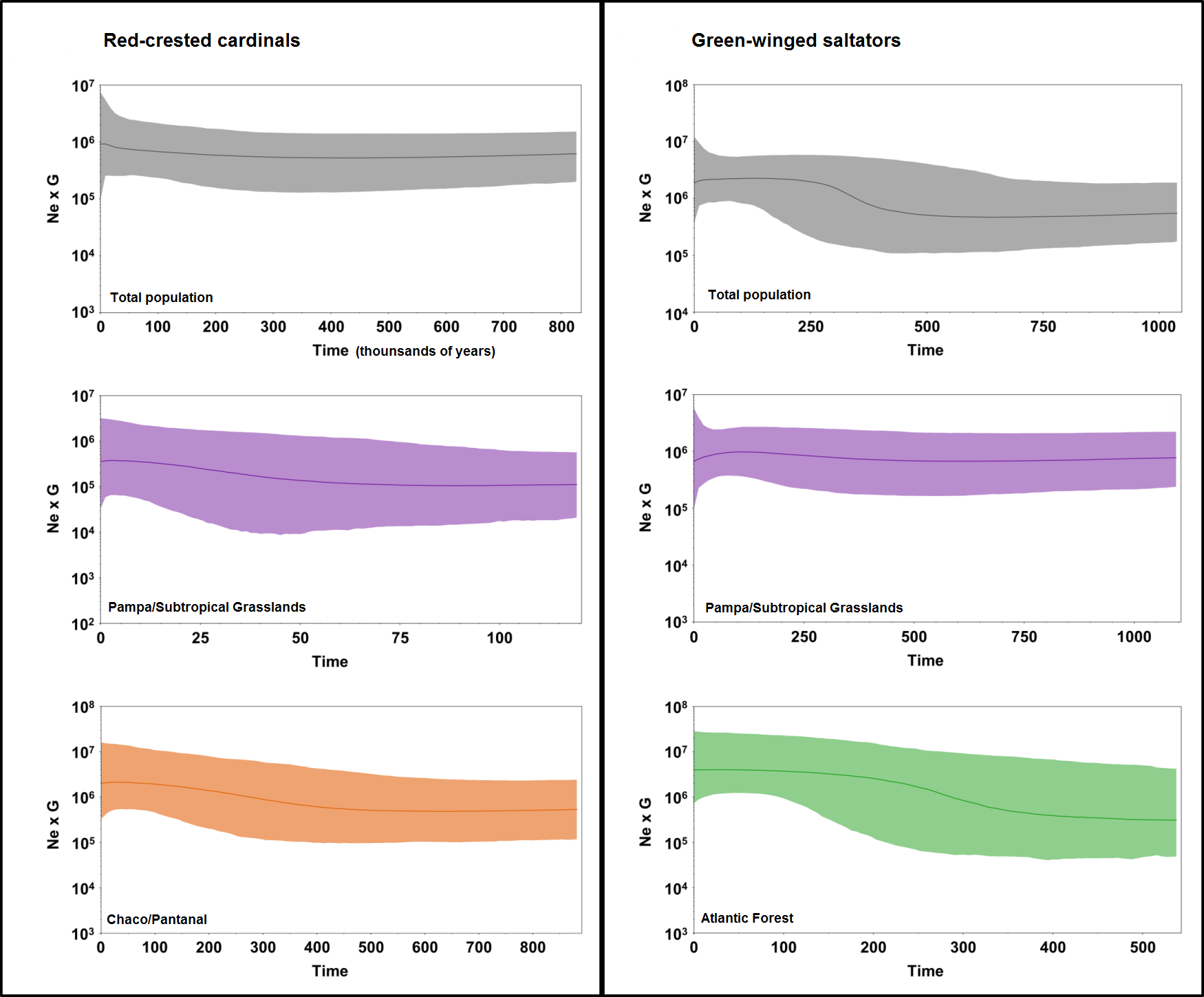

Supplement: Supplementary Figure 1 — Bayesian skyline plot for both species. [file Image_1.TIFF]
