# Supplementary material for: A Preliminary Assessment of the Potential Health and Genetic Impacts of Releasing Confiscated Passerines Into the Wild: A Reduced-Risk Approach
Source: Front Vet Sci. 2021 Oct 11;8:679049. doi: 10.3389/fvets.2021.679049 (PMC8542797; doi:10.3389/fvets.2021.679049)
Supplement: Supplementary Table 4 — Genetic diversity indices. [file Data_Sheet_1.PDF]

Supplementary Table 4. Standard genetic diversity indices for red-crested cardinals and green-winged saltators.

	**Source**	**Sample size**	**N Loc**	**Segregating sites**	**N Haplot**	**Haplotype diversity[1]**	**Nucleotide diversity[1]**	**Tajima's D[2]**	**Fu's Fs[2]**
red-crested cardinals	Pampa/SG	43	8	7	6	0.41 (0.09)	0.0006 (0.0005)	**-1.82**	**-3.18**
	Chaco/Pantanal	13	3	25	9	0.92 (0.06)	0.0066 (0.0038)	-0.86	-1.02
	Atlantic Forest	3	1	2	3	1.00 (0.27)	0.0014 (0.0014)	0.00	-1.22
	Total	59	12	30	14	0.60 (0.07)	0.0022 (0.0014)	**-2.17**	**-4.76**
green-winged saltators	Pampa/SG	45	9	26	14	0.86 (0.04)	0.0058 (0.0032)	-0.69	-1.24
	Chaco/Pantanal	3	2	2	2	0.67 (0.31)	0.0017 (0.0017)	0.00	1.06
	Atlantic Forest	27	7	26	18	0.93 (0.04)	0.0042 (0.0025)	**-1.79**	**-11.37**
	Total	75	18	43	27	0.90 (0.03)	0.0055 (0.0031)	**-1.59**	**-10.65**

N Loc: Number of localities; N Haplot: Number of haplotypes; [1]Values in parenthesis are the standard deviation; [2]Values in bold have a P-value <0.05 for Tajima's D and <0.02 for Fu's Fs.
